# Clinical and Biochemical Correlates of Parathyroid Gland Burden in Patients Undergoing Parathyroidectomy for Secondary Hyperparathyroidism: A Retrospective Observational Study

**DOI:** 10.3390/jcm15072564

**Published:** 2026-03-27

**Authors:** Muhammet Fatih Keyif, Sabahattin Destek

**Affiliations:** 1 Department of General Surgery, Faculty of Medicine, Bolu Abant Izzet Baysal University, Bolu 14030, Türkiye; 2 Department of General Surgery, Faculty of Medicine, Uskudar University, Istanbul 34662, Türkiye; sebahattindestek@yahoo.com

**Keywords:** secondary hyperparathyroidism, parathyroidectomy, parathyroid gland burden, ultrasonography

## Abstract

**Background/Objectives**: Secondary hyperparathyroidism (SHPT) is a common and clinically significant complication of advanced chronic kidney disease and may require surgical intervention when medical therapy fails. This study aimed to evaluate the association between parathyroid gland burden, defined by gland number and size parameters, and biochemical disease severity in patients undergoing parathyroidectomy for SHPT, and to assess the concordance between preoperative imaging findings and intraoperative observations. Although parathyroid gland enlargement is a hallmark of SHPT, the clinical relevance of parathyroid gland number and overall gland burden in relation to biochemical disease severity and the accuracy of preoperative imaging remains incompletely defined. **Methods**: This single-center, retrospective observational study included adult patients who underwent parathyroidectomy for secondary hyperparathyroidism between January 2015 and December 2020. Demographic, clinical, laboratory, imaging, intraoperative, and histopathological data were analyzed. Parathyroid gland burden was assessed based on gland number, largest gland diameter, and total gland burden. Associations between gland morphology and biochemical parameters were evaluated using correlation analyses and multivariable logistic regression. Agreement between preoperative imaging and intraoperative findings was assessed using diagnostic performance metrics, contingency analysis, and the weighted kappa coefficient. **Results**: A total of 101 patients were included. Patients with three or more enlarged parathyroid glands had significantly higher preoperative parathyroid hormone and alkaline phosphatase levels, higher serum phosphorus levels, and lower calcium and vitamin D levels (all *p* < 0.05). Total gland burden and largest gland diameter were positively correlated with parathyroid hormone and alkaline phosphatase levels. In multivariable analysis, higher parathyroid hormone levels, longer dialysis duration, and vitamin D deficiency were independently associated with high gland burden. Preoperative imaging demonstrated moderate agreement with intraoperative findings (weighted kappa = 0.46; 95% CI, 0.29–0.63). Separate evaluation of imaging modalities showed that both ultrasonography and scintigraphy had relatively high sensitivity but limited specificity for detecting extensive gland involvement. **Conclusions**: In patients undergoing parathyroidectomy for secondary hyperparathyroidism, increased parathyroid gland burden is associated with greater biochemical disease severity. Preoperative imaging shows limited concordance with intraoperative findings and should be interpreted cautiously, particularly in the presence of multiglandular disease. These findings support the integration of morphological parameters into comprehensive preoperative assessment while highlighting the need for larger prospective, multicenter studies with standardized imaging protocols and long-term follow-up.

## 1. Introduction

Secondary hyperparathyroidism (SHPT) is a common and clinically significant complication of advanced chronic kidney disease (CKD), characterized by profound disturbances in mineral and bone metabolism [1,2]. In patients with end-stage renal disease, impaired calcium, phosphorus, and vitamin D homeostasis leads to sustained elevation of parathyroid hormone (PTH) levels and progressive parathyroid gland hyperplasia. Beyond its skeletal effects, SHPT is associated with cardiovascular complications, vascular calcification, neuromuscular symptoms, and increased morbidity and mortality [3].

The pathophysiology of SHPT is driven primarily by phosphate retention, hypocalcemia, and reduced levels of active vitamin D. In response to these biochemical alterations, the parathyroid glands initially undergo diffuse hyperplasia; however, with disease progression, nodular hyperplasia may develop. Nodular transformation is particularly relevant because it is often associated with resistance to medical therapy and relative autonomy of PTH secretion, contributing to persistent biochemical abnormalities [4,5].

Medical management remains the first-line treatment for SHPT and typically includes dietary modification, phosphate binders, active vitamin D analogues, and calcimimetic agents. Nevertheless, a subset of patients develops refractory disease characterized by persistently elevated PTH levels and ongoing clinical manifestations despite optimized medical therapy. In such cases, surgical intervention in the form of parathyroidectomy represents an effective and well-established therapeutic option [6,7]. In patients with advanced or medically refractory disease, surgery remains the definitive treatment for achieving biochemical control and preventing long-term complications associated with severe hyperparathyroidism.

Accurate preoperative assessment of parathyroid gland number, size, and distribution is crucial for optimal surgical planning. Neck ultrasonography and parathyroid scintigraphy are widely used for preoperative localization; however, their ability to reliably reflect intraoperative findings remains variable [8]. This limitation is particularly relevant in secondary hyperparathyroidism, where multiglandular hyperplasia is the predominant pathological pattern and may reduce the diagnostic accuracy of imaging modalities. Moreover, the relationship between parathyroid gland morphology—particularly gland number and size—and the biochemical severity of SHPT has not been fully elucidated. This gap limits the ability to anticipate surgical complexity and tailor operative strategies based on preoperative data.

Although previous studies have examined associations between PTH levels and parathyroid gland volume, comprehensive evaluations incorporating both the number of involved glands and gland dimensions in relation to clinical and laboratory parameters are limited [9,10]. In addition, the degree of concordance between preoperative imaging findings and intraoperative observations in SHPT patients remains insufficiently characterized. Furthermore, whether morphological indicators of parathyroid disease burden correlate with biochemical severity and clinical parameters in patients requiring surgery remains an area of ongoing investigation.

The aim of the present study was to evaluate the association between the number and size of involved parathyroid glands and clinical as well as laboratory findings in patients undergoing parathyroidectomy for secondary hyperparathyroidism. A secondary objective was to assess the agreement between preoperative imaging modalities and intraoperative findings. By providing a detailed characterization of parathyroid gland burden in relation to biochemical parameters, this study seeks to contribute clinically relevant data to the preoperative evaluation and surgical management of patients with SHPT.

## 2. Materials and Methods

### 2.1. Study Design and Ethical Approval

This study was designed as a single-center, retrospective observational study conducted at Sancaktepe Şehit Prof. Dr. İlhan Varank Training and Research Hospital, a tertiary referral center for endocrine and renal surgery. Patients who underwent parathyroidectomy for secondary hyperparathyroidism between January 2015 and December 2020 were retrospectively identified from institutional electronic medical records.

Secondary hyperparathyroidism (SHPT) was defined as persistently elevated serum parathyroid hormone (PTH) levels (>800 pg/mL or >9 times the upper limit of normal) in patients with advanced chronic kidney disease (CKD stage 5 or stage 5D) receiving dialysis therapy, accompanied by disturbances in calcium–phosphorus metabolism and resistance to optimized medical treatment. Indications for surgery were based on institutional clinical practice and international guideline recommendations, including persistently elevated PTH levels despite optimized medical therapy with phosphate binders, vitamin D analogues, and calcimimetic agents.

The study protocol was reviewed and approved by the Institutional Ethics Committee of Sancaktepe Şehit Prof. Dr. İlhan Varank Training and Research Hospital (Approval No: 2021/152; Date: 9 June 2021). The study was conducted in accordance with the ethical principles of the Declaration of Helsinki. Owing to the retrospective nature of the study and the use of anonymized patient data, the requirement for informed consent was waived by the ethics committee.

### 2.2. Study Population and Patient Selection

Patients who underwent surgical treatment for secondary hyperparathyroidism were retrospectively identified from institutional electronic medical records. Adult patients (≥18 years) with a diagnosis of secondary hyperparathyroidism related to chronic kidney disease who underwent parathyroidectomy and had complete preoperative, intraoperative, and postoperative data were eligible for inclusion.

Secondary hyperparathyroidism was distinguished from primary and tertiary hyperparathyroidism based on clinical history, biochemical profile, and dialysis status. Patients with hypercalcemia suggestive of primary hyperparathyroidism, those with autonomous PTH secretion following kidney transplantation suggestive of tertiary hyperparathyroidism, and patients with prior parathyroid surgery were excluded.

**Figure 1:**
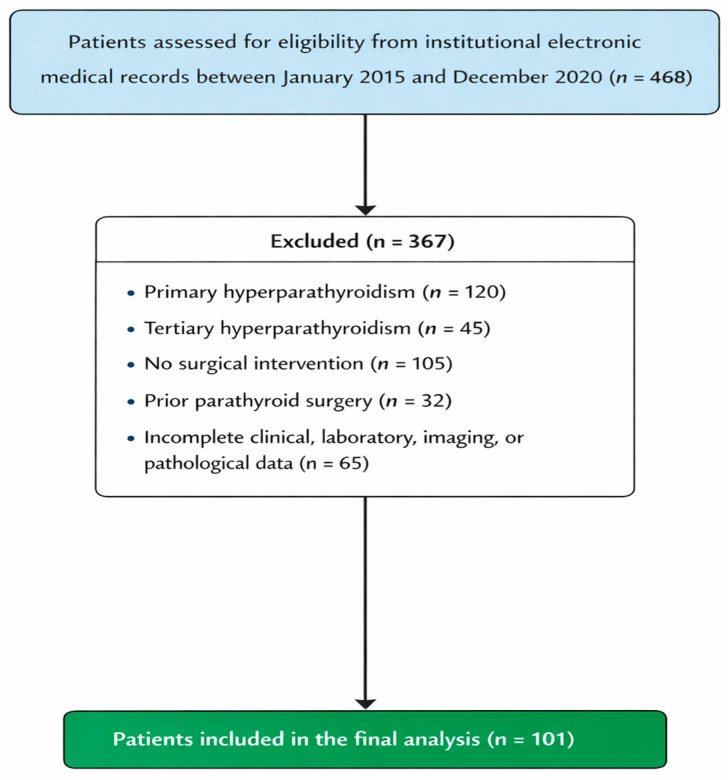
Flow diagram of patient selection and study inclusion process.

Patients were excluded if they had primary or tertiary hyperparathyroidism, did not undergo surgical intervention, had a history of prior parathyroid surgery, or had incomplete clinical, laboratory, imaging, or pathological data. After application of inclusion and exclusion criteria, a total of 101 patients were included in the final analysis. The patient selection process is summarized in Figure 1.

### 2.3. Clinical and Demographic Data

Demographic data including age and sex were recorded for all patients. Clinical variables collected included dialysis modality (hemodialysis or peritoneal dialysis), duration of dialysis, and the presence of comorbid conditions such as diabetes mellitus, hypertension, and cardiovascular disease. The indication for surgical intervention was documented in all patients and was defined as medically refractory secondary hyperparathyroidism despite optimized medical therapy, including dietary measures, phosphate binders, vitamin D analogues, and calcimimetic agents.

### 2.4. Laboratory Data Collection

Preoperative laboratory parameters were obtained from routine blood tests performed within 7 days prior to surgery. In patients receiving hemodialysis, blood samples were collected immediately before the dialysis session in order to minimize dialysis-related biochemical fluctuations.

Recorded laboratory parameters included total serum calcium, phosphorus, alkaline phosphatase, parathyroid hormone (PTH), and 25-hydroxyvitamin D levels. Serum calcium values represent total serum calcium measurements. Albumin-corrected calcium values were not routinely available in all patients; therefore, albumin correction could not be applied in the analysis. Accordingly, calcium values reported in the study correspond to uncorrected total serum calcium concentrations. The calcium–phosphorus product was calculated for each patient using standard formulas.

Parathyroid hormone levels were measured using a second-generation intact PTH immunoassay (iPTH assay) routinely used in the hospital laboratory for the evaluation of secondary hyperparathyroidism in patients with chronic kidney disease. This assay detects biologically active PTH (1–84) as well as certain circulating PTH fragments, which may lead to partial overestimation of biologically active hormone levels in patients with advanced chronic kidney disease. However, this assay represents the standard laboratory method used in routine clinical practice at our institution during the study period.

Postoperative laboratory values, including serum calcium, phosphorus, alkaline phosphatase, and parathyroid hormone levels, were recorded at the time of hospital discharge (typically postoperative day 2–4) to assess early biochemical outcomes following surgery.

Information regarding preoperative vitamin D supplementation was not consistently available in the retrospective records and therefore could not be included in the analysis. This limitation was addressed in the study limitations section.

The reference ranges used in the institutional laboratory during the study period were as follows: parathyroid hormone (PTH) 15–65 pg/mL, total serum calcium 8.6–10.2 mg/dL, phosphorus 2.5–4.5 mg/dL, alkaline phosphatase 40–130 IU/L, and 25-hydroxyvitamin D 20–50 ng/mL.

### 2.5. Imaging Assessment

All patients underwent preoperative parathyroid imaging as part of routine clinical evaluation. Imaging modalities included neck ultrasonography and technetium-99m sestamibi parathyroid scintigraphy performed according to institutional diagnostic protocols.

Ultrasonographic examinations were conducted by experienced radiologists specialized in endocrine imaging using high-resolution linear transducers (e.g., GE Healthcare, Chicago, IL, USA), and the number of suspected enlarged parathyroid glands as well as the maximal diameter of each identified gland were recorded in the radiology reports.

For the purposes of this study, an enlarged parathyroid gland was defined as a gland with a maximal diameter ≥ 10 mm on imaging or intraoperative assessment.

Scintigraphic imaging was performed using technetium-99m sestamibi (e.g., Mallinckrodt Pharmaceuticals, St. Louis, MO, USA) with planar imaging, and when clinically indicated, additional single-photon emission computed tomography (SPECT) imaging was obtained to improve anatomical localization.

Imaging reports were retrospectively reviewed to extract the number of enlarged glands and their maximal diameters. In cases where both ultrasonography and scintigraphy were performed, findings were interpreted in conjunction with the radiological reports. Imaging results were recorded independently from intraoperative findings to allow an unbiased comparison between preoperative localization studies and surgical exploration.

### 2.6. Surgical Technique and Intraoperative Evaluation

All surgical procedures were performed by experienced endocrine surgeons using standard operative techniques for the treatment of secondary hyperparathyroidism. The choice of surgical procedure, including subtotal parathyroidectomy or total parathyroidectomy with or without autotransplantation, was determined intraoperatively based on gland morphology, surgeon judgment, and established surgical practice. During surgery, systematic bilateral neck exploration was performed to identify all parathyroid glands. The number of identified glands was recorded, and the maximal diameter of each excised gland was measured intraoperatively using a sterile surgical ruler immediately after gland removal. These measurements were documented in the operative reports. Total parathyroid gland burden was calculated as the sum of the maximal diameters of all identified glands. Although gland volume or weight may more accurately reflect total parathyroid tissue mass, diameter measurements were consistently available in operative documentation and therefore were used as a standardized surrogate measure of gland burden in this retrospective analysis. All intraoperative findings, including gland number, location, and morphological characteristics, were systematically recorded.

### 2.7. Histopathological Examination

All excised parathyroid specimens were submitted for routine histopathological examination. Tissue samples were evaluated by experienced pathologists who were blinded to the clinical, laboratory, and imaging findings of the patients. Histopathological evaluation was performed according to established diagnostic criteria for parathyroid disease, and the specimens were classified as diffuse hyperplasia or nodular hyperplasia. Diffuse hyperplasia was defined by uniform proliferation of parathyroid chief cells involving the entire gland, whereas nodular hyperplasia was characterized by nodular cellular proliferation with partial architectural distortion. Histopathological findings were recorded for each patient and incorporated into the final dataset for descriptive analysis.

### 2.8. Outcome Measures

The primary outcomes of this study were the morphological characteristics of the parathyroid glands, including the number of involved glands, the maximal diameter of the largest gland, and the calculated total gland burden. Total gland burden was defined as the sum of maximal diameters of all identified parathyroid glands and was used as a surrogate indicator of the overall extent of parathyroid hyperplasia. Secondary outcomes included the relationship between gland morphology and preoperative biochemical parameters, early postoperative biochemical changes following parathyroidectomy, and the level of agreement between preoperative imaging findings and intraoperative observations. These outcomes were selected to explore the association between anatomical gland characteristics and biochemical disease severity, as well as to evaluate the diagnostic performance of preoperative imaging modalities in predicting operative findings.

### 2.9. Statistical Analysis

All statistical analyses were performed using IBM SPSS Statistics for Windows, version 23.0 (IBM Corp., Armonk, NY, USA). Continuous variables were assessed for normality using the Shapiro–Wilk test together with visual inspection of histograms and Q–Q plots. Normally distributed continuous variables were expressed as mean ± standard deviation, whereas non-normally distributed variables were presented as median and interquartile range. Categorical variables were summarized as frequencies and percentages.

Comparisons between independent groups were performed using the independent samples *t*-test for normally distributed continuous variables and the Mann–Whitney U test for non-normally distributed variables. Paired comparisons of preoperative and postoperative biochemical measurements were conducted using the paired samples *t*-test or the Wilcoxon signed-rank test as appropriate.

Categorical variables were compared using the chi-square test or Fisher’s exact test when expected cell counts were less than five. Associations between parathyroid gland morphological parameters, including gland number, maximal gland diameter, and total gland burden, and biochemical variables were evaluated using Spearman’s rank correlation coefficient due to the non-normal distribution of several laboratory parameters.

Multivariable logistic regression analysis was conducted to identify factors associated with high parathyroid gland burden, defined as the presence of three or more enlarged parathyroid glands. Variables included in the regression model were selected based on clinical relevance and results of univariable analyses.

The results were presented as adjusted odds ratios with 95% confidence intervals. Model adequacy was assessed using the Hosmer–Lemeshow goodness-of-fit test, and multicollinearity among predictors was evaluated using variance inflation factors (VIF).

The diagnostic performance of preoperative imaging for identifying high parathyroid gland burden was assessed by calculating sensitivity, specificity, positive predictive value, and negative predictive value. Agreement between preoperative imaging findings and intraoperative observations beyond chance was evaluated using the weighted kappa coefficient. In addition, a sensitivity analysis was performed in which the number of parathyroid glands was treated as a continuous variable rather than a categorical variable (<3 vs. ≥3 glands). The overall direction and statistical significance of the associations between gland morphology and biochemical parameters remained consistent, supporting the robustness of the primary analyses. All statistical tests were two-sided, and a *p* value less than 0.05 was considered statistically significant.

## 3. Results

A total of 101 patients who underwent parathyroidectomy for secondary hyperparathyroidism were included in the study. The mean age of the cohort was 52.3 ± 10.9 years, and 58 patients (57.4%) were male. The median duration of dialysis was 72 months (range, 18–192 months). Hemodialysis was the predominant dialysis modality, accounting for 92.1% of patients. The prevalence of comorbidities in the study population included diabetes mellitus in 40.6% of patients, hypertension in 86.1%, and cardiovascular disease in 32.7%. The baseline demographic and clinical characteristics of the study population are summarized in Table 1.

Preoperative laboratory parameters are presented in Table 2. The median preoperative parathyroid hormone (PTH) level was 1450 pg/mL (IQR, 980–2210), and the median alkaline phosphatase (ALP) level was 320 IU/L (IQR, 235–465). Mean serum calcium and phosphorus levels were 9.1 ± 0.7 mg/dL and 6.3 ± 1.4 mg/dL, respectively. The median 25-hydroxyvitamin D level was 14.0 ng/mL (IQR, 9.0–22.0).

Parathyroid gland characteristics assessed by preoperative imaging and intraoperative exploration are summarized in Table 3. Preoperative imaging identified a median of three enlarged glands (range, 1–4), whereas intraoperative exploration revealed a median of four glands (range, 3–5). The proportion of patients with three or more enlarged glands was 73.3% according to imaging findings and 90.1% according to intraoperative assessment.

To explore the relationship between gland number and biochemical parameters, patients were stratified according to the number of glands identified intraoperatively (<3 vs. ≥3 glands). Comparisons of preoperative laboratory parameters between these groups are shown in Table 4. Patients with ≥3 glands had significantly higher median PTH levels compared with those with fewer glands (1600 vs. 980 pg/mL, *p* = 0.003). Similarly, ALP levels were higher in the ≥3 gland group (340 vs. 220 IU/L, *p* = 0.006). Serum phosphorus levels were also significantly elevated in patients with ≥3 glands (*p* = 0.018), whereas serum calcium and vitamin D levels were significantly lower in this group (*p* = 0.041 and *p* = 0.002, respectively).

Correlation analyses between gland morphological parameters and biochemical variables are presented in Table 5. Total gland burden demonstrated a moderate positive correlation with PTH levels (r = 0.48, 95% CI: 0.31–0.61, *p* < 0.001) and ALP levels (r = 0.41, 95% CI: 0.24–0.56, *p* < 0.001). Similarly, the largest gland diameter showed a positive correlation with PTH levels (r = 0.42, 95% CI: 0.25–0.56, *p* < 0.001) and serum phosphorus levels (r = 0.29, 95% CI: 0.10–0.45, *p* = 0.003). The number of glands identified intraoperatively also showed a positive correlation with PTH and ALP levels, although correlations with serum calcium were weaker. Overall, these correlations correspond to modest effect sizes, explaining approximately 9–23% of the variance in biochemical parameters (r^2^ range: 0.09–0.23). Sensitivity analysis treating gland number as a continuous variable yielded results consistent with the primary analyses, supporting the robustness of the observed associations.

Early postoperative biochemical parameters recorded at the time of hospital discharge are shown in Table 6. Median PTH levels decreased markedly from 1450 pg/mL preoperatively to 85 pg/mL postoperatively (*p* < 0.001). Significant reductions were also observed in serum calcium and phosphorus levels following surgery (both *p* < 0.001). In contrast, alkaline phosphatase levels showed minimal early postoperative change.

Multivariable logistic regression analysis was performed to identify independent factors associated with high parathyroid gland burden (≥3 glands). As presented in Table 7, higher preoperative PTH levels (OR 1.45, 95% CI 1.10–2.12; *p* = 0.012), longer dialysis duration (OR 1.18, 95% CI 1.02–1.45; *p* = 0.031), and vitamin D deficiency (OR 3.20, 95% CI 1.05–11.10; *p* = 0.041) were independently associated with increased gland burden. Alkaline phosphatase did not reach statistical significance in the multivariable model (*p* = 0.070). Model diagnostics indicated adequate model fit (Hosmer–Lemeshow test *p* = 0.41), and variance inflation factors were <2 for all variables, suggesting no significant multicollinearity.

The agreement between preoperative imaging findings and intraoperative observations for identifying patients with three or more enlarged glands is presented in Table 8. The weighted kappa coefficient was 0.46 (95% CI: 0.29–0.63), indicating moderate agreement between imaging and surgical findings. Preoperative imaging demonstrated a sensitivity of 81.3% and a specificity of 54.5% for detecting high parathyroid gland burden. While imaging showed a relatively high positive predictive value, the negative predictive value remained limited, suggesting that imaging studies may underestimate the presence of additional hyperplastic glands in some patients.

Table 9 presents the diagnostic performance of individual imaging modalities for detecting high parathyroid gland burden. Scintigraphy demonstrated slightly higher sensitivity compared with ultrasonography, whereas specificity remained limited for both modalities. When both imaging techniques were interpreted together, the overall diagnostic performance remained comparable to the combined imaging results reported in Table 8.

The cross-tabulation of imaging findings and intraoperative observations is shown in Table 10. The weighted kappa coefficient was 0.46 (95% CI: 0.29–0.63), indicating moderate agreement between preoperative imaging and intraoperative assessment.

## 4. Discussion

In this study, we comprehensively evaluated the associations between parathyroid gland morphology and clinical as well as biochemical parameters in patients undergoing parathyroidectomy for secondary hyperparathyroidism. The principal findings indicate that increased parathyroid gland burden—defined by both the number of involved glands and gland size parameters—is closely associated with biochemical disease severity. In addition, we observed only moderate agreement between preoperative imaging findings and intraoperative observations, highlighting the limitations of imaging-based assessment in this patient population.

One of the key findings of the present study is that patients with three or more intraoperatively identified parathyroid glands exhibited significantly higher preoperative parathyroid hormone and alkaline phosphatase levels. This observation suggests that gland number is not merely an anatomical descriptor but may reflect the underlying biological activity and severity of secondary hyperparathyroidism. While previous studies have primarily focused on parathyroid gland volume or weight as markers of disease burden, the number of involved glands has received comparatively less attention [11,12].

From a clinical perspective, an increased number of hyperplastic glands may represent a more advanced stage of disease characterized by diffuse or nodular hyperplasia affecting multiple glands. This widespread involvement may contribute to reduced responsiveness to medical therapy and increased surgical complexity [13,14]. Therefore, gland number may serve as a practical and clinically meaningful parameter when evaluating disease severity and anticipating surgical challenges, although it should not be interpreted in isolation.

In addition to gland number, both the largest gland diameter and total gland burden demonstrated significant positive correlations with parathyroid hormone and alkaline phosphatase levels. These findings support the concept that the total amount of hyperplastic parathyroid tissue, rather than the presence of a single dominant gland, plays a central role in driving biochemical activity in secondary hyperparathyroidism [15].

Total gland burden, calculated as the sum of maximal gland diameters, may offer a more comprehensive representation of disease extent than individual gland measurements. Clinically, this parameter may be particularly relevant during preoperative assessment, as it could assist surgeons in anticipating the need for more extensive exploration or favoring subtotal or total parathyroidectomy over limited resection. Nevertheless, given the retrospective nature of this study, these observations should be regarded as hypothesis-generating rather than prescriptive [16]. It should also be noted that this parameter was derived from intraoperative measurements; therefore, its application in preoperative risk stratification remains exploratory and requires validation in prospective studies.

Elevated serum phosphorus levels were significantly associated with increased gland number and gland burden in this cohort. This finding is consistent with the established pathophysiological role of phosphate retention in stimulating parathyroid cell proliferation and parathyroid hormone secretion in chronic kidney disease. Persistent hyperphosphatemia may act as a chronic proliferative stimulus, contributing to progressive parathyroid hyperplasia and resistance to medical therapy [17].

In clinical practice, these results underscore the importance of sustained phosphorus control in patients with advanced chronic kidney disease. Poor long-term phosphorus management may not only worsen biochemical control but also promote more extensive parathyroid gland involvement, potentially increasing the likelihood of surgical intervention. Notably, the biochemical differences observed between gland-burden strata in this study (e.g., PTH levels of approximately 980 vs. 1600 pg/mL) are clinically relevant when considered in the context of guideline-based treatment targets for secondary hyperparathyroidism, such as those proposed by KDIGO, which recommend maintaining PTH levels within several-fold of the upper normal limit in dialysis patients.

Vitamin D deficiency emerged as an independent factor associated with high parathyroid gland burden in multivariable analysis. This association aligns with the known suppressive effect of vitamin D on parathyroid hormone secretion and parathyroid cell proliferation. Prolonged vitamin D deficiency may contribute to continuous parathyroid stimulation, favoring the development of diffuse and nodular hyperplasia [18,19].

However, the interpretation of this relationship requires caution. Vitamin D deficiency may represent both a contributor to and a consequence of severe secondary hyperparathyroidism, and a bidirectional relationship is likely [20]. Therefore, the present findings do not support a causal inference regarding vitamin D deficiency and gland burden, nor do they suggest that vitamin D supplementation alone could prevent surgical disease progression.

The independent association between longer dialysis duration and increased parathyroid gland burden highlights the cumulative nature of secondary hyperparathyroidism. Prolonged exposure to metabolic derangements associated with chronic kidney disease may facilitate the transition from diffuse to nodular hyperplasia, leading to reduced sensitivity to medical therapy [21,22]. Clinically, this finding suggests that patients with long dialysis vintage may represent a higher-risk subgroup with respect to extensive parathyroid involvement. While this observation may aid in risk stratification, it does not establish an optimal timing for surgical intervention, which remains a complex decision requiring individualized clinical judgment.

Our analysis demonstrated only moderate agreement between preoperative imaging and intraoperative findings in identifying patients with high parathyroid gland burden. Although imaging modalities showed relatively high sensitivity, their specificity was limited, indicating a tendency toward misclassification in some cases [11,23]. The weighted kappa coefficient further confirmed only moderate concordance between imaging-based assessment and surgical findings, suggesting that preoperative imaging alone may not reliably identify the full extent of multiglandular hyperplasia. These results reinforce the notion that preoperative imaging should be regarded as an adjunct rather than a definitive determinant of surgical strategy in secondary hyperparathyroidism.

Several factors may contribute to this discordance between imaging and operative findings, including small gland size, ectopic gland locations, and the presence of diffuse multiglandular hyperplasia, which is typical of secondary hyperparathyroidism. In such settings, imaging modalities may successfully identify dominant glands while underestimating the full extent of glandular involvement.

Quantitatively, the discordance between imaging and intraoperative findings was reflected by the diagnostic performance metrics. Preoperative imaging demonstrated a sensitivity of 81.3% but a specificity of only 54.5% for identifying patients with ≥3 enlarged glands. Furthermore, the negative predictive value was low (28.6%), indicating that a substantial proportion of patients classified as having fewer glands on imaging were found to have multiglandular involvement during surgery. These findings highlight that imaging tends to underestimate the extent of glandular disease in secondary hyperparathyroidism.

Early postoperative biochemical assessment revealed marked reductions in parathyroid hormone, calcium, and phosphorus levels, reflecting the effectiveness of parathyroidectomy in achieving immediate biochemical control [24,25]. In contrast, alkaline phosphatase levels exhibited minimal early change, likely reflecting ongoing bone remodeling processes that normalize over a longer time frame. Interestingly, the correlation between the largest gland diameter and serum calcium levels was weak and not statistically significant. This finding may reflect the complex regulation of calcium homeostasis in dialysis patients, which is influenced not only by parathyroid hormone activity but also by factors such as dialysate calcium concentration, calcium-containing phosphate binders, and variations in dialysis prescriptions.

Collectively, the findings of this study suggest that parathyroid gland number and overall gland burden may provide clinically meaningful information regarding disease extent in secondary hyperparathyroidism, complementing conventional biochemical assessment. In routine practice, surgical referral and planning are largely guided by persistent biochemical abnormalities despite optimized medical therapy; however, our results indicate that morphological parameters may help contextualize biochemical severity and identify patients with more widespread parathyroid involvement. Awareness of a high gland burden phenotype may assist surgeons in anticipating operative complexity and potential discordance between preoperative imaging and intraoperative findings, thereby supporting operative preparedness rather than dictating a specific surgical strategy. Importantly, given the moderate agreement observed between imaging and intraoperative assessment, morphological findings should be interpreted cautiously and integrated with clinical presentation, laboratory data, dialysis duration, and surgical judgment. Overall, parathyroid gland morphology appears best positioned as a complementary component of a comprehensive, multidisciplinary preoperative evaluation rather than a standalone decision-making tool. Importantly, the associations observed in this study should be interpreted within the broader clinical context of secondary hyperparathyroidism management. Although increased gland number and gland burden were correlated with biochemical disease severity, these morphological parameters should not be considered independent indications for surgical intervention. Rather, they may provide additional contextual information alongside established clinical criteria such as persistent biochemical abnormalities, symptom burden, and failure of optimized medical therapy.

Following these observations, the additional analyses performed for individual imaging modalities provided further insight into the diagnostic performance of preoperative localization studies. When ultrasonography and scintigraphy were evaluated separately, both modalities demonstrated relatively high sensitivity for detecting patients with extensive gland involvement, whereas specificity remained limited. As presented in Table 9, scintigraphy showed slightly higher sensitivity than ultrasonography, although the overall diagnostic performance of both techniques remained comparable. These findings are consistent with previous reports indicating that imaging accuracy is lower in secondary hyperparathyroidism compared with primary hyperparathyroidism, largely because multiglandular hyperplasia is the predominant pathological pattern. In this setting, imaging techniques may successfully identify dominant glands but may fail to accurately characterize the full extent of glandular involvement.

To further evaluate the agreement between imaging findings and surgical exploration, a contingency analysis comparing imaging-based classification and intraoperative gland burden was performed. Although imaging demonstrated high positive predictive values for identifying patients with extensive gland involvement, the negative predictive value was limited. This pattern suggests that imaging studies may underestimate the presence of additional hyperplastic glands in a substantial proportion of patients. Consequently, reliance solely on imaging findings for determining the extent of surgery may lead to incomplete assessment of glandular disease. These results reinforce the clinical principle that preoperative imaging should primarily be used to assist localization and operative planning, whereas comprehensive intraoperative exploration remains essential for accurately determining the number and extent of hyperplastic parathyroid glands in patients with secondary hyperparathyroidism.

Taken together, these additional analyses highlight the complementary roles of biochemical assessment, morphological evaluation, and imaging studies in the preoperative management of secondary hyperparathyroidism. While imaging contributes valuable anatomical information, its limitations in detecting multiglandular disease underscore the continued importance of surgical expertise and intraoperative evaluation in guiding definitive management.

Several limitations of this study should be acknowledged when interpreting the findings. First, the retrospective and single-center design inherently limits external validity and may introduce selection bias, as patient inclusion depended on the availability and completeness of clinical records. In addition, patients included in the study represent a selected subgroup of individuals with secondary hyperparathyroidism who underwent surgical treatment after failure of medical therapy, which may further limit the generalizability of the findings to the broader population of patients with chronic kidney disease. Moreover, the absence of a control group consisting of medically managed patients further limits comparative interpretation and external applicability. Although this design reflects real-world clinical practice, the results may not be fully generalizable to other centers with different patient populations, surgical expertise, or referral patterns. Furthermore, the study period spanned several years, during which temporal changes in clinical management, imaging practices, and surgical decision-making may have occurred, potentially introducing additional heterogeneity into the dataset.

Second, postoperative evaluation was confined to early biochemical parameters obtained at hospital discharge. Consequently, long-term outcomes such as recurrent hyperparathyroidism, sustained biochemical control, changes in bone mineral density, fracture risk, cardiovascular events, or survival could not be assessed. Given the chronic and progressive nature of secondary hyperparathyroidism, the lack of longitudinal follow-up limits the ability to draw conclusions regarding the durability of surgical outcomes or the prognostic significance of gland burden over time. Moreover, postoperative complications such as hypocalcemia and hungry bone syndrome, as well as the proportion of patients achieving predefined biochemical targets or biochemical cure criteria, were not systematically available in the retrospective records and therefore could not be analyzed. In addition, the marked imbalance between patients with <3 glands and those with ≥3 glands may have affected the stability of the logistic regression estimates, and the multivariable findings should therefore be interpreted with caution. This imbalance also increases the risk of model overfitting and reduces the robustness of regression-based inferences.

Third, preoperative imaging studies were performed as part of routine clinical care rather than according to a standardized research protocol. Variability in imaging modality selection, operator experience, and reporting practices may have contributed to interobserver variability and influenced the observed level of agreement between imaging and intraoperative findings. Additionally, not all patients underwent both ultrasonography and scintigraphy, which may have affected the comparative assessment of imaging performance. Although additional analyses evaluating individual imaging modalities were performed, the retrospective nature of the dataset limited the ability to apply uniform imaging protocols or standardized measurement criteria across all patients. Furthermore, imaging evaluations were based on radiology reports rather than blinded re-assessment of raw imaging data, which may underestimate interobserver variability and affect the generalizability of the findings.

Fourth, morphological assessment of parathyroid glands was based primarily on gland number and maximal diameter measurements. More detailed quantitative parameters, such as gland weight, volume, or histomorphometric features, were not available and therefore could not be incorporated into the analysis. As a result, total gland burden may have been incompletely characterized, and subtle differences in parathyroid tissue mass could not be fully captured. Furthermore, diameter-based estimation of gland burden may not fully reflect the true volumetric extent of hyperplastic parathyroid tissue. The use of the sum of maximal diameters as a surrogate measure represents a pragmatic but non-validated approach and may introduce measurement-related bias. More accurate volumetric or weight-based assessments would provide a more reliable representation of true gland burden. In addition, the definition of high gland burden based on ≥3 glands represents a clinically practical but somewhat arbitrary threshold that requires further validation in future studies.

Finally, although multivariable analysis was performed to adjust for clinically relevant factors, the possibility of residual confounding cannot be excluded. In particular, biochemical parameters in dialysis patients may be influenced by multiple clinical factors, including dialysis prescription, dialysate calcium concentration, phosphate binder use, and variations in vitamin D therapy. Because detailed information regarding these treatment-related variables was not consistently available, their potential influence on biochemical measurements could not be fully accounted for in the present analysis. In particular, the lack of data on phosphate binder use, calcimimetic therapy, dialysate calcium concentration, and detailed vitamin D supplementation represents an important limitation. In addition, potential interaction effects between selected variables, such as vitamin D status and PTH levels or dialysis duration and patient age, were not formally modeled because of the limited sample size and the risk of model overfitting. Taken together, these limitations indicate that the present findings should be interpreted as associative rather than causal. Future multicenter, prospective studies with standardized imaging protocols, comprehensive morphometric assessment, and long-term follow-up are warranted to validate and expand upon these observations.

## 5. Conclusions

In conclusion, increased parathyroid gland burden, reflected by both gland number and size parameters, was associated with markers of greater biochemical disease severity in patients undergoing parathyroidectomy for secondary hyperparathyroidism. Preoperative imaging demonstrated only moderate concordance with intraoperative findings, and separate modality-based analysis further highlighted the limited specificity of imaging for characterizing multiglandular disease. These findings suggest that morphological parameters may complement biochemical assessment during preoperative evaluation; however, their clinical utility should be interpreted cautiously given the retrospective design, potential selection bias related to inclusion of only surgically treated patients, and the absence of long-term outcome data. In addition, the use of diameter-based gland burden as a non-validated surrogate and the presence of group imbalance may further limit the robustness and generalizability of the findings. Accordingly, gland morphology should be considered as a supportive component of comprehensive clinical assessment rather than an independent determinant of surgical decision-making. Future prospective, multicenter studies incorporating standardized imaging protocols and volumetric gland assessment are warranted to validate these findings and clarify their clinical applicability.

## Figures and Tables

**Table 1:** Baseline Demographic and Clinical Characteristics of the Study Population (n = 101).

Variable	Value
Age (years), mean ± SD	52.3 ± 10.9
Male sex, n (%)	58 (57.4)
Dialysis duration (months), median (min–max)	72 (18–192)
Hemodialysis, n (%)	93 (92.1)
Diabetes mellitus, n (%)	41 (40.6)
Hypertension, n (%)	87 (86.1)
Cardiovascular disease, n (%)	33 (32.7)

SD, standard deviation.

**Table 2:** Preoperative Laboratory Parameters.

Parameter	Value
Calcium (mg/dL), mean ± SD	9.1 ± 0.7
Phosphorus (mg/dL), mean ± SD	6.3 ± 1.4
Calcium–phosphorus product, mean ± SD	57.3 ± 14.6
Alkaline phosphatase (IU/L), median (IQR)	320 (235–465)
Parathyroid hormone (pg/mL), median (IQR)	1450 (980–2210)
25-OH vitamin D (ng/mL), median (IQR)	14.0 (9.0–22.0)

SD, standard deviation; IQR, interquartile range.

**Table 3:** Parathyroid Gland Characteristics Based on Imaging and Intraoperative Findings.

Variable	Imaging	Intraoperative
Number of glands, median (range)	3 (1–4)	4 (3–5)
≥3 glands, n (%)	74 (73.3)	91 (90.1)
Largest gland diameter (mm), mean ± SD	15.2 ± 5.1	17.0 ± 5.6
Total gland burden (mm), mean ± SD	48.0 ± 16.2	56.4 ± 18.0

SD, standard deviation. Enlarged gland was defined as a parathyroid gland with a maximal diameter ≥ 10 mm.

**Table 4:** Preoperative Laboratory Parameters According to Intraoperatively Identified Parathyroid Gland Number.

Parameter	<3 Glands (n = 10)	≥3 Glands (n = 91)	*p* Value
PTH (pg/mL), median (IQR)	980 (720–1320)	1600 (1050–2350)	0.003
ALP (IU/L), median (IQR)	220 (180–310)	340 (245–480)	0.006
Calcium (mg/dL), mean ± SD	9.4 ± 0.6	9.0 ± 0.7	0.041
Phosphorus (mg/dL), mean ± SD	5.4 ± 1.1	6.4 ± 1.4	0.018
25-OH vitamin D (ng/mL), median (IQR)	20.0 (14.0–28.0)	13.0 (8.0–20.0)	0.002

PTH, parathyroid hormone; ALP, alkaline phosphatase; SD, standard deviation; IQR, interquartile range. Group comparisons were performed using the Mann–Whitney U test for non-normally distributed variables and the independent samples *t*-test for normally distributed variables.

**Table 5:** Correlation Between Gland Morphology and Laboratory Parameters.

Variable	PTH	ALP	Calcium	Phosphorus
Largest gland diameter	r = 0.42 (95% CI: 0.25–0.56), *p* < 0.001	r = 0.35 (95% CI: 0.17–0.50), *p* < 0.001	r = −0.18 (95% CI: −0.36–0.01), *p* = 0.071	r = 0.29 (95% CI: 0.10–0.45), *p* = 0.003
Total gland burden	r = 0.48 (95% CI: 0.31–0.61), *p* < 0.001	r = 0.41 (95% CI: 0.24–0.56), *p* < 0.001	r = −0.22 (95% CI: −0.39–−0.03), *p* = 0.027	r = 0.33 (95% CI: 0.15–0.48), *p* < 0.001
Number of glands	r = 0.39 (95% CI: 0.21–0.54), *p* < 0.001	r = 0.31 (95% CI: 0.12–0.47), *p* = 0.001	r = −0.10 (95% CI: −0.29–0.10), *p* = 0.318	r = 0.24 (95% CI: 0.05–0.41), *p* = 0.015

PTH, parathyroid hormone; ALP, alkaline phosphatase; CI, confidence interval.

**Table 6:** Preoperative and Early Postoperative Biochemical Parameters.

Parameter	Preoperative	Postoperative	*p* Value
PTH (pg/mL), median (IQR)	1450 (980–2210)	85 (52–140)	<0.001
Calcium (mg/dL), mean ± SD	9.1 ± 0.7	7.8 ± 0.8	<0.001
Phosphorus (mg/dL), mean ± SD	6.3 ± 1.4	4.2 ± 1.0	<0.001
ALP (IU/L), median (IQR)	320 (235–465)	330 (250–430)	0.041

PTH, parathyroid hormone; ALP, alkaline phosphatase.

**Table 7:** Multivariable Logistic Regression Analysis for High Parathyroid Gland Burden (≥3 Glands).

Variable	Adjusted OR (95% CI)	*p* Value
Parathyroid hormone (per 500 pg/mL increase)	1.45 (1.10–2.12)	0.012
Dialysis duration (per 12 months)	1.18 (1.02–1.45)	0.031
Vitamin D deficiency (<20 ng/mL)	3.20 (1.05–11.10)	0.041
Alkaline phosphatase (per 100 IU/L increase)	1.22 (0.98–1.62)	0.070

OR, odds ratio; CI, confidence interval.

**Table 8:** Agreement Between Imaging and Intraoperative Findings for High Gland Burden (≥3 Glands).

Measure	Value
Sensitivity (%)	81.3
Specificity (%)	54.5
Positive predictive value (%)	92.6
Negative predictive value (%)	28.6
Weighted kappa coefficient (95% CI)	0.46 (0.29–0.63)

PPV, positive predictive value; NPV, negative predictive value.

**Table 9:** Diagnostic Performance of Preoperative Imaging Modalities for Detecting High Parathyroid Gland Burden (≥3 Glands).

Imaging Modality	Sensitivity (%)	Specificity (%)	Positive Predictive Value (%)	Negative Predictive Value (%)
Ultrasonography	78.6	52.0	90.1	27.5
Scintigraphy (Tc-99m sestamibi)	83.5	57.1	93.2	31.4
Combined imaging *	81.3	54.5	92.6	28.6

* Combined imaging refers to the interpretation of ultrasonography and scintigraphy together when both modalities were available. High gland burden was defined as the presence of ≥3 enlarged glands. Enlarged gland was defined as a gland with a maximal diameter ≥10 mm. PPV, positive predictive value; NPV, negative predictive value.

**Table 10:** Cross-Tabulation of Preoperative Imaging and Intraoperative Findings for High Parathyroid Gland Burden.

	Intraoperative < 3 Glands	Intraoperative ≥ 3 Glands	Total
Imaging < 3 glands	6	21	27
Imaging ≥ 3 glands	4	70	74
Total	10	91	101

High gland burden was defined as the presence of ≥3 enlarged glands. Enlarged gland was defined as a gland with a maximal diameter ≥ 10 mm. Weighted kappa coefficient = 0.46 (95% CI: 0.29–0.63).

## Data Availability

The data presented in this study are available from the corresponding author upon reasonable request. The data are not publicly available due to institutional and ethical restrictions related to patient confidentiality.

## Abbreviations

ALP	Alkaline phosphatase
CKD	Chronic kidney disease
CI	Confidence interval
IQR	Interquartile range
NPV	Negative predictive value
OR	Odds ratio
PTH	Parathyroid hormone
PPV	Positive predictive value
SD	Standard deviation
SHPT	Secondary hyperparathyroidism
